# A qualitative exploration of cultural safety in nursing from the perspectives of Advanced Practice Nurses: meaning, barriers, and prospects

**DOI:** 10.1186/s12912-022-00960-9

**Published:** 2022-07-04

**Authors:** Jacqueline Pirhofer, Johannes Bükki, Mojtaba Vaismoradi, Manela Glarcher, Piret Paal

**Affiliations:** 1 Institute of Nursing Science and Practice, Paracelsus Medical University, Strubergasse 21, 5020 Salzburg, Austria; 2 Diakonie-Klinikum Stuttgart, Rosenbergstrasse 38, 70176 Stuttgart, Germany; 3 Faculty of Nursing and Health Sciences, Nord University, 8049 Bodø, Norway

**Keywords:** Cultural safety, Nursing, Power, Healthcare, Discrimination, Equity, Caregiving

## Abstract

**Background:**

Cultural safety requires healthcare professionals and organisations to improve healthcare, facilitate patient access to healthcare, and achieve equity within the workforce.

**Methods:**

This ethnomethodological study, which consisted of two phases, explored the concept of cultural safety from the perspective of Advanced Practice Nurses. Semi-structured interviews and the nominal group technique were used to prioritise challenges related to cultural safety, identify barriers to clinical practise and assess educational needs. The data collected was subjected to thematic analysis.

**Results:**

Nurses working in Austria, Germany and Switzerland were recruited (*N* = 29). Accordingly, the phenomenon of cultural safety in health care is not generally known and there is little prior knowledge about it. The most frequently discussed themes were communication difficulties, lack of knowledge, the treatment of people with insufficient language skills and expectations of treatment goals and outcomes, which can lead to conflicts and accusations of unequal treatment due to diverse cultural backgrounds.

**Conclusion:**

Diverse cultures are encountered in German-speaking healthcare settings. Cultural safety is also about healthcare staff, as nurses with different socialisations encounter prejudice, discrimination and racism. Although the issue of power was not discussed, academic nurses were willing to make an effort to change. Only a minority were aware that lasting change requires challenging one’s own cultural structures and adapted behaviours, rather than pushing for the mere acquisition of cultural competence. Organisations were encouraged to introduce self-reflection sessions and provide better access to translation services to improve equity and support nurses.

## Background

The right to health was recognised as a human right in the 1966 International Covenant on Economic, Social, and Cultural Rights. According to the United Nations Office of the High Commissioner for Human Rights (OHCHR), healthcare services must be culturally appropriate and acceptable [1]. The International Council of Nurses (ICN) Code of Ethics states: “Inherent in nursing is a respect for human rights, including cultural rights.” [2]. As nursing care is delivered within a social context, it is affected by healthcare contexts and policies. The importance of culturally appropriate and acceptable healthcare is steadily increasing with the increasing pluralism, diversity, and complexity of ethnic and cultural groups [3].

Health equity aims to eliminate disparities and achieve optimal health. Existing guidelines mention the discrimination of people who do not have territorial or social access to health care services. In this context, the culturally acceptable meaning of access is still debated [4]. Access to healthcare services is affected by geography, ethnicity, sex, and socioeconomic status. Globally, in-country mobility and rural–urban migration patterns add to growing diversity. Hence, accessible healthcare is an issue that is not only available in indigenous populations but also among people with different migration patterns and their descendants. Subpopulations have specific risks and exposures that must be addressed in healthcare. In the United States, the National Institutes of Health (NIH) introduced standards to enhance culturally and linguistically appropriate services (CLAS) to be adapted to any healthcare setting to improve equity and eliminate healthcare disparities [5]. The new European Agenda for Culture of the European Council calls on research on cultural crossovers, assessing the impacts of culture in different fields, including health and well-being [6].

Even in countries where cultural safety accreditation standards and policies exist, they may not necessarily be implemented [7]. Perry et al. (2015) have demonstrated that during the clinical assessment of patients from culturally and linguistically diverse backgrounds, nurses frequently rely on patients’ poor language skills, use family members or patients’ friends as interpreters, and/or do not discuss the cultural aspects of health for fear of offending patients [8]. Healthcare providers establish interventions for their individual and professional definitions of ‘appropriate’ care. There is an ingrained assumption that healthcare professionals are solely responsible for this because of their expertise. Discussions on patient autonomy, partnership, and informed consent are ongoing. However, the premise of equal treatment for all patients is insufficient, as it simply ignores existing differences [9]. To ensure high-quality care, detailed biography and needs/resource assessments are required. Patients need space for self-definition, including their respective life experiences and experiences of the care process [10]. Accordingly, governments have committed to improving access to healthcare, enriching health service delivery, improving the quality of service and patient safety, and increasing the efficiency and coordination of care [11].

Safe healthcare services should be adequate in several dimensions such as culture. Culture is a highly context-dependent concept explained as “a patterned behavioural response that develops over time as a result of imprinting the mind through social and religious structures and intellectual and artistic manifestations.” [12]. Unless this definition of culture is acknowledged in healthcare, socio-cultural practices that are ethically, socio-politically, economically questionable, and even oppressive are perpetuated [13, 14]. It consists of all strong indicators for seeking novel approaches to heed culturally safe patient care. Curtis et al. (2019) thoroughly discussed why cultural safety rather than cultural competency is required to achieve health equity. Based on their findings, they proposed the following definition of ‘cultural safety’:

*Cultural safety deals with the balance of power between professional caregivers and people receiving care. Accordingly, professional caregivers must recognise that a person’s dignity and right to self-determination have to be acknowledged and prioritised; address their own biases, attitudes, assumptions, stereotypes, prejudices, structures, and characteristics that may affect the quality of care; and engage in self-reflection. Healthcare organisations and authorities need to be held accountable for providing culturally safe care, as defined by patients and their communities, and as measured through progress towards achieving health equity. Cultural safety requires healthcare professionals and their associated organisations to influence healthcare to reduce bias and achieve equity within the workforce and working environment* [15].

Wilson et al. (2022) critically analysed cultural safety education and its translation into practice, focusing on indigenous health education programs. They demonstrated that even in countries where cultural safety is a well-established concept, the change in practice is complex, requiring leadership for a multi-system approach, critical pedagogy, and commitment by nurses. Educational programs for building consciousness are required to have an impact. Education is a change agent; however, it is only one part of the complex and interdependent change process to achieve culturally safe patient care. Understanding how nurses practice cultural safety in local contexts is essential to forge changes at the individual, educational, and organizational levels [16].

Comprehensively assessing cultural safety may improve our understanding of these phenomena, identify areas for improvement, and promote cultural re-engineering strategies that improve patient safety [17, 18]. In this study, we explored the culturally safe practices of ADVANCED PRACTICE NURSES (APN) in the German language context. According to the ICN, an advanced practice nurse is “a registered nurse who has acquired the expert knowledge base, complex decision-making skills, and clinical competencies for expanded practice, the characteristics of which are shaped by the context or country in which [she or he] is credentialed to practice.” [19]. Their focus is on direct clinical practice, addressing the health needs of populations, individuals, and families with additional expertise in health promotion and disease prevention [20]. In German-speaking countries, the integrative model of APN proposed by Hamric and Hanson [21] is widely used to describe APN core competencies, namely guidance and coaching, counselling, evidence-based practice, leadership, and collaboration [22]. It is important to understand that the educational model is an agreement between tertiary institutions to promote and professionalise nursing practice. The role of the APN is not recognised by any regulatory or government body in the respective countries [23–27]. In Austria, Germany and Switzerland, nurses can obtain a Master’s degree, but there are no career paths or clearly defined roles for APNs. Unlike APNs, the Registered Nurse designation (RN) is a governmental attempt to register practicing nurses, and since there is no re presentative body for nurses, there are no requirements other than to join the register. Compared to RNs or Advanced Practice Registered Nursing (APRN) roles, APNs are academically educated nurses, thus by fulfilling an expanded nursing role and performing broad competencies in healthcare, they may be able to create awareness and serve as role models for cultural safety. Therefore, this study aims to explore the concept of cultural safety from an APNs’ point of view by identifying existing barriers, opportunities, and practices.

## Methods

This qualitative exploration consisted of two phases: semi-structured interviews to develop new knowledge of the research phenomenon and a nominal group meeting to discuss the findings, collect more detailed data, and validate the information power of the data collected in Phase 1.

### Design

This ethnomethodological study helps improve our understanding of the construction of order and reality through the production and interpretation of meanings from the perspective of acting persons [28]. People act as a matter of routine, and the goal of ethnomethodology is to explain people’s behaviours and actions as evidence. This approach sets out to use theoretical resources and empirical research to understand processes and, as a second step, contributes to the change in context [29].

### Phase 1. Semi-structured interviews

To gain new insights into cultural safety, which is not an established theory, adequate information power was considered in the sampling strategy [30]. Interviews were conducted between November 2020 and March 2021 with nurses working in Austria, Germany or Switzerland. Second-year Master’s students (120 ETCS) and APNs with Master’s degrees were recruited. For Phase 1, the interviewer (JP) tried to recruit only students through personal contact. Since participants could not be reached in this way, we decided to expand the sample to include APNs with university degrees. Our failure to recruit students was partly due to Covid-19-related stressors and constraints and partly due to a lack of interest in the research topic.

Extended, in-depth, and specialised nursing practice takes place through direct interactions with/among individuals/families or groups [31, 32]. APNs are expected to engage in sociocultural reflection, adopt new concepts, and rethink their cultural practices. The participants differed in age, professional experience, and specialty. Thus, this study benefitted from a broad range of experiences. Data regarding their religious and ethnic backgrounds were obtained but were not reported because of anonymity and confidentiality.

Participants were recruited via email using the purposive sampling method. The invitation contained an explanation, research questions, the purpose of data collection, and a declaration of consent. If interested, participants were asked to return the signed information sheet. An appointment was made for the interview, which was conducted through online video conferences. This turned out to be extremely helpful, especially since access to nursing staff from other countries was facilitated, and research could also be carried out in accordance with the pandemic rules. To ensure that no third parties were present, the interviewer switched on her camera and showed the interviewees their premises. Casual introductory conversations were held before the sound and video recordings. All interviews were conducted in German. The interviews lasted between 21 and 51 min (Table 1).

**Table 1:** Duration of interviews and characteristics of interview participants

Code	Duration, minute	Gender	Age	Setting	Work experience in years
P1	35:21	Male	26	Hospital ward	2,5
P2	23:27	Female	29	Hospital ward	4
P3	51:34	Male	33	Hospital ward	7
P4	21:02	Female	25	Hospital ward	2,5
P5	36:30	Female	30	Hospital ward	7
P6	31:54	Female	26	Hospital ward	3
P7	32:32	Female	25	Hospital ward	3
P8	24:33	Female	43	Outpatient area	25
P9	33:32	Male	48	Outpatient area	25
P10	26:39	Female	30	Outpatient area	1,5

The interviews were transcribed *verbatim* immediately after each interview [33]. Longer pauses for breath have been placed in brackets and integrated into the text (1–3 s pause). Striking stresses have been written in bold. Striking sounds were written in brackets, e.g. (mhmm) and (aah). Striking, non-linguistic processes are marked with asterisks, e.g. *raises his voice* and *laughs*. Data collection and analysis were performed concurrently. The collected data were subjected thematic analysis [34]. Thematic analysis is a flexible research strategy as no linear data analysis process is assumed. This type of analysis suited our study because there have been no previous studies on this topic from the German-speaking context. The first author, who conducted the interviews and transcribed them, created and managed the initial codes through open coding using the MaxQDA software. Codes were constantly compared, sorted, and classified based on their similarities and differences. Themes and subthemes were developed iteratively by comparing codes that were reviewed and refined by the research team. The authors collaborated during the data analysis process until the themes were finalised [34, 35].

#### Rigor and trustworthiness

Two experts in qualitative thematic analysis (JP and PP) assessed the study’s compliance with the guidelines for establishing trustworthiness. To ensure credibility and reliability, a sample of transcripts was independently reviewed and coded by JP and PP. A consensus debriefing was conducted to explore the relationships between the interview and the background context to increase reflexivity and validate findings. For credibility, individual transcripts, along with a brief report of the study findings, were sent back to the participants. None of the respondents made any requests for change, indicating that our findings reflected their perspectives. The bilingual authors ensured the validity of the translation of the outcomes from German to English for publication.

### Phase 2. Nominal group meeting

Phase 1 served as the main phase of data collection for this study. However, Phase 2 was required to present, discuss and verify the data collected in the previous phase. It also served to check the validity of the data collected in Phase 1. Therefore, the nominal group technique was used. The nominal group technique (NGT) is a useful method for capturing priorities in health care and considers themes identified by the whole group [36]. The participants were second-year Master’s students (Table 2) who were asked to prioritise challenges related to cultural safety, name their importance, explore barriers to clinical practice and identify their educational needs based on the data collected in Phase 1. Students who had attended the lecture on cultural encounters in nursing were recruited and presented with the results of the previous phase and then asked to share their views.

**Table 2:** Characteristics of the NGT participants

Gender	Setting	Work experience in years
Female	Intensive Care Unit	10
Female	Outpatient Care	6
Female	Center of Practical Guidance	10
Female	Hospital Ward	11
Female	Hospital Ward	0,5
Female	Intensive Care Unit	15
Female	Hospital Ward	20
Female	Hospital Ward	2
Female	Hospital Ward	10
Female	Hospital Ward	6
Female	Hospital Ward	14
Female	Parental Leave	0
Female	Outpatient Care	5
Female	Hospital Ward	2
Female	Hospital Ward	2
Male	Special Area	13
Male	Intensive Care Unit	4
Male	Hospital Ward	13
Male	Hospital Ward	6

A Padlet dashboard (www.padlet.com) was created as part of the instructional session and the link was sent to the participants. They were able to use the comment function to document and record all ideas. The process took approximately 15 min to complete. During this process, participants were also able to ask questions about the topic. Multiple responses were provided. It addressed the educational needs of the APNs to improve the existing curriculum. Participants were asked three questions related to cultural safety: 1. What factors stand out to me, which are particularly challenging? 2. What are the prospects for transformation in nursing care? 3. What aspects should be integrated into nursing education? To discern patterns, similar codes were grouped and summarised in a table. Next, they were analysed using thematic analysis [34] and presented as a tree diagram combining the study findings from Phases 1 and 2.

## Results

### Participants

A total of 29 students and graduates participated in this study consisting of ten students and graduates in Phase 1 and 19 students in Phase 2. The majority (78%) of the participants were women. Their mean work experience was 7.9 years (range = 0–25). Of all the participants, 62% (*n* = 18) worked in inpatient wards, 17% (*n* = 5) in the outpatient setting, 10% (*n* = 3) in the intensive care unit, one in central practice coordination, and one was on parental leave.

### Phase 1: semi-structured interviews

Three main themes were developed during the data analysis process: ‘meaning of cultural safety’, ‘barriers to cultural safety’, and ‘prospects of cultural safety’. The themes and their related subthemes are described below, using direct quotations from the participants (Table 3).

**Table 3:** The summary of themes and subthemes

Theme	Subtheme
Meaning of cultural safety	Impact on the patients Self-assessment of APNs Prospective development
Barriers to cultural safety	Discrimination Importance of communication Visiting relatives Gender roles Nutrition Fears Time Religion Conflicts
Prospects of cultural safety	Possibilities of nursing staff Possibilities of the organizations Proactive involvement of family members

#### Meaning of cultural safety

Cultural safety was common in healthcare settings, but there was little prior knowledge about it: *if I were to hear (aah) cultural safety, in everyday care and everyday life, and in general, I would first say it is not yet a widespread topic or terminology. This is not a taken-for-granted terminology. This is not the present conceptualisation of everyday nursing care.* (Participant 3, Line 34).

A uniform understanding of the phenomenon of cultural safety was reported, with cornerstones on individuality and the need for security, regardless of origin, culture, or other demographic determinants. Its primary goal was to provide the best possible individualised care. *Therefore, under cultural safety, I understand that no matter what a person is like, in terms of sexual orientation, occupation, and socioeconomic status, is safe in this environment*. (P1, L. 60).

The acceptance of both society and personnel was inevitable and any marginalisation had to be hindered: *so, that he [the patient] can just live out the way, he is and move around in this can move within this protected framework without having to make cuts.* (P1, L. 60).

##### Impact on the patients

The situation of the patients in the hospital should never be recognised as neutral. Patients might experience pain, psychological overload, or even trauma. Nursing care should focus on cultural and personal needs that positively influence therapeutic relationships. Physical discomfort often occurs along with emotional discomfort. Therefore, feelings of insecurity prevailed before the establishment of a nursing relationship.

A commonly shared opinion was that cultural safety influenced achieving the therapy goals, well-being, quality of life, recovery process, and psyche of the patient: *I could imagine, they feel taken care of. More understood. Respected, even integrated. So, or so perceived. They feel more comfortable, and the feeling of comfort is also a safety aspect, and therefore, contributes to recovery.* (P8, L. 60).

##### Self-assessment of APNs

*Do you provide culturally safe care?* Nine out of ten participants answered, *No*. They tried it, but never reached the perfect condition. *So, my answer to this question can be balanced; I think, I do my work 100 percent perfect all the time. I would strongly argue that no nurse can be 100 percent culturally safe.* (P3, L. 38).

*I try. However, I make mistakes that I am not aware of. So, like patronising the patient, yes, he wants it that way. That happens. However, this is simply unconscious. I would say that I would never do it purposely. However, it happens in everyday life and through habits.* (P7, L. 50)

Cultural safety was difficult to achieve because it was not an endpoint, but a continuous process. Continuous training was highlighted because basic education was not sufficient: *I do not really know much about it, I mean, of course, in the school you have heard about the differences of (mhmm) certain things, but I do not think I am really informed about it, no.* (P6, L. 46).

##### Prospective development

Some participants stated that currently no progress was under way regarding cultural safety: *I do not think, no, that it will develop in the direction now, because nursing has quite different construction sites.* (P1, L. 32).

Current research has indicated novel approaches to generate a benefit for nursing, but the transfer of knowledge into practice is slow. Special attention must be paid to this sector to meet future care demands, *and we must massively upgrade and improve it. I mean, with migration, that is, what we are experiencing all around us. This will certainly be a challenge for the next few years.* (P9, L. 42).

#### Barriers to cultural safety

Different aspects of each culture are often transformed automatically into stereotypes by nurses to make their work easier. *In principle, a difficult patient is not difficult; he simply has needs that could not be met until now. You just must talk to them out of it, or they need something different in general.* (P10, L. 34).

It is typically triggered by cultural background, making it difficult to process information. Due to staff shortages, nursing staff are increasingly reaching imposed limits and barriers. An adjustment should take place by which nurses and patients must approach each other to generate suitable solutions for the future: *But I think a certain form of adaptation, I would wish for on both sides. The adaptation of a hospital takes place in the form of interpreter services or special meals* … (P8, L. 42).

##### Discrimination

It was stated that prejudices were shaped or formed by situations in the past and by the media: *I think that often you get into this predicament that you might say something not so sensitive, (aah) although you do not want to discriminate, but (aah) it can happen in the sense of a faux pas.* (P3, L. 28).

This discourse occurred only when people from diverse cultural backgrounds were involved. Explicit discrimination rarely occurred, but they confessed that devaluation in the form of jokes occurred in various settings. "Balkan syndrome" as a unique experience of pain and a louder and more pronounced suffering was specified: *Yes. [3–5 s pause] Either way, of course, there is the classic saying ‘Balkan Syndrome’. If you go out now, for example, in the direction of a patient with an ex-Yugoslav background. (aah) Yes, I do not like it. When I hear something like this, I always point it out to my colleagues.* (P2, L. 36).

Despite some participants denying any racism, others reported racist behaviours, such as a complete lack of appreciation for certain patients. Prejudices towards people with diverse cultural backgrounds were mentioned: *For me, it is also always difficult to comprehend when it is in practice that other caregivers (mhmm) condemn or change their behaviours because of that–towards the patients.* (P7, L. 22).

##### Importance of communication

Verbal communication is a vital tool for establishing contact with patients, building relationships, receiving information, and communicating, *which is exceedingly difficult. I consider communication to be one of the highest assets in professional nursing. You cannot communicate; they say so nicely.* (P3, L. 30).

A big caring issue was nurses rejecting patients: *Quite often, the language barrier was a huge problem. Many nurses are already averse or [takes a long pause for breath] from the patient alone when a language barrier is present.* (P7, L. 36).

This caused patients sometimes not to receive care they were entitled to. *Often, the patients are simply left out of the rounds by the doctors.* (P7, L. 42).

The language barrier created a heteronomy that was not conducive to patient care: *I also see it critically that if you live in a country and get sick and cannot express yourself, then it’s an enormously dependent relationship and then it is somehow difficult to guarantee medical care.* (P8, L. 42).

##### Visiting relatives

Families expressed their appreciation by visiting an ill family member. For certain ethnic groups, such visits were of high value: *if you look at ethnic origin [3–5 s pause], with southerners, family is always a very present theme.* (P3, L. 34).

*The whole family always wants to be present, preferably so that they can show affection, so that they can feel connected to the patient, so that they can provide for better recovery and positively favour the whole thing. Moreover, they have high conflict potential.* (P3, L. 42)

This was a burden when too many relatives were present in the patient’s room. A negative aspect was the increased noise that prevailed in rooms or wards. Others expressed an understanding towards visitations and criticised the disapproving attitude of nursing staff: *Families like this do not come with bad intentions; it is about a community about celebrating something normal. (ahm) I have always found that it is very beneficial for patients. And exactly, found it a pity when the nursing staff then spoke so deprecatingly.* (P8, L. 38).

##### Gender roles

Gender roles and body image are complex entities in many cultures, and individual considerations of sociocultural aspects are considered important. *Of course, there is also the whole relationship between body image and body care. One must also always pay attention to the fact that one does not necessarily bring a male colleague to a Muslim woman in the room, or you must agree to this beforehand. The same is true, but also *vice versa*. Men from diverse cultural backgrounds can also be helped by other men. This is something you must pay attention to.* (P2, L. 32).

##### Nutrition

Eating was seen as a cultural sign of becoming well again: *food plays a crucial role because in our society (long breath), when it now goes towards the end of life, food (aah) no longer has such a significant importance as in other cultures. In other cultures, it often seems to me that they try to continue to give the patient food, and if he eats, then that is a good sign. Since then, he has become healthy again. You also notice cultural differences.* (P2, L. 32).

The caregivers tended to associate special dietary requirements, such as the rejection of pork, negatively. This seemed burdensome, adding to the workload. *For example, the food without pork, oh God, now you must order the food again. It is always an effort that is seen and less positive.* (P7, L. 40).

##### Fears

The participants had a basic understanding of patients’ fears, including the fear of making situations in the hospital difficult for people with diverse cultural backgrounds; *when I imagine how a patient feels, who may not be in the hospital very often, and who does not understand the language, and especially now at Corona times, when no one is allowed in the hospital, and then maybe she does not know how to use her cell phone because she is very old, then it is a disaster, I can imagine.* (P1, L. 48).

*It also helped me to see certain situations with different eyes. That is, not everything is the way it comes across. However, people behave in such a way because they are afraid of the reactions of another person. They tried to protect themselves.* (P5, L. 22)

##### Time

Several reasons including time for not being able to provide culturally safe care were mentioned: *[3–5 s pause, unintelligible] It is often difficult to take time with a patient (aah) who may not understand me. Yes, this makes this difficult to achieve.* (P2, L. 54).

*Thus, there is more time. Time for the patient. Especially in acute hospitals (mhmm), the time to deal with it. I think a lot about palliative situations or of deceased patients when relatives come. It is just the case that, if they need something, they should get in touch. However, you do not clarify in advance how things are done or what their wishes are.* (P7, L. 22)

##### Religion

The participants acknowledged the need to learn about others’ religions. *When I think back to Germany, where the Muslim community is very widely represented, one can also be in lively contact with this community. During a hospital stay, you learn an enormous amount, and then you also develop this sensitivity for certain subtleties accordingly, which is now, for example, in connection with religion.* (P3, L. 22).

Religion was set as it only represented one part of a person; *despite religious affiliation, a reduction in religion is not desirable. A human being should always be recognised as an individual. Therefore, every person, (aah), has his or her character; hence, he or she must be considered an individual, and this also applies to religion. Religion is only a part, a fraction of which, what a human being is from its whole, or the total.* (P3, L. 24).

##### Conflicts

Cultural diversities led to conflicts that, under certain circumstances, should be addressed: *it is important, especially when certain conflicts blossom, to address them directly and to try to present one’s interests or one’s view clearly and to act accordingly in a patient-oriented manner, that is, according to the motto patient first, and accordingly to reflect on one’s own potential conflicts and on them within the team with a critical view of one’s personality or one’s interests.* (P3, L. 26).

Due to diverse personalities, team cultures were variable, and conflicts might arise. Therefore, self-reflection was deemed essential: *And (aah) on the other hand, it is incredibly important to reflect on oneself at the end of the day and to take the best from these conflicts and try to grow from them afterwards. It is part of your feedback to each other within the team at some point.* (P3, L. 26).

#### Prospects of cultural safety

Staff, employers, and relatives were considered relevant actors in improving cultural safety.

##### Possibilities of nursing staff

It was essential to put aside one’s impartiality toward foreign cultures and to act as a professional. Professional and culturally safe care presupposes certain soft skills; *therefore, we need greater sensitivity, understanding, and empathy. That is a process. [3–5 s pause], we are on the right path. I am positive about this. However, you can do much more.* (P5, L. 50).

Quality management is important to continuously improve cultural safety. A valuable tool was feedback, *which was a good keyword in this context [long pause for breath] because it offers the possibility to make one or another person more aware.* (P3, L. 26).

The patient’s feedback was also a suitable medium: *And there I plead that the patient also gives some feedback and lets us say there are various mechanisms that you can use, like an (aah) feedback sheet after a hospital stay*. (P3, L. 28).

Four out of ten nurses mentioned reflection as an important perspective in achieving culturally safe care: *And (aah) on the other hand, it is incredibly important that you reflect on yourself at the end of the day and, in the case of these conflicts, to take the best of them and try to grow a little bit afterward. It is part of that that your feedback is to each other within the team at some point. (P3, L. 26) Every nurse should be concerned with themselves: How do I want the patient to be treated? Think about how I would like to be treated?* (P10, L. 34).

Close observation and adaptation were also stated: *I think in our profession we should always be able to observe well. Moreover, they have certain adaptability. Regardless of what our own interests and values and principles are, which sometimes may not be compatible or correlate with one another.* (P3, L. 24).

Empathy is a central pillar of a successful patient relationship: Understanding, I think, is a crucial point. (mhmm) [long pause for breath] *clear throat* I find the topic so difficult because it should be simple and easy. (P4, L. 50).

There needed to be awareness or education regarding cultural aspects. *What certainly needs to happen is that all caregivers or medical professionals have a little more input about something like that, because I can only talk about myself now, I only know the standard things.* (P6, L. 53).

The relationship between the patient and caregiver was important during the hospital stay, *and the relationship is, for me let us say, the fundamental aspect of nursing care*. (P9, L. 28).

A detailed assessment at the beginning of each stay was a crucial step for all further measures: *Yes, take a history, also illuminate the cultural background, where he comes from, and how he grew up. (ah) [Long pause for breath] What are the family circumstances? A detailed assessment is important.* (P2, L. 42).

*History taking and the initial interview are always a good start for me, so to get to know, no matter which person is in front of me now. I would make a difference, but it is about gathering information about the person and the person and documenting things that are important to him or her (long pause for breath), and then it is also a matter of disseminating that further within the team. For this purpose, communication must be possible.* (P8, L. 44)

Prejudices should be put aside on the part of the caregiver to approach the person in a completely unprejudiced manner to establish a communicative relationship. *I believe that if the patient trusts us, it is important that we all (aah) so that we try to understand. (a) We cannot expect everybody to understand us.* (P5, L. 52).

Some respondents stated that cultural characteristics had no role in establishing a professional relationship, while others claimed the opposite: *it would certainly be professional if you knew the culture or the patient’s origin and if you simply responded to the individual needs. However, this often gets lost in everyday life and in stress through the automatic process that we simply know.* (P7, L. 48).

Acknowledging individuality might even make work with people of diverse cultures more rewarding. *And then I realised, gosh, how much I enjoy it when people like me, when I like the people like, when they build relationships*. (P9, L. 30).

The participants’ curiosity about foreign cultures was considered an advantage to increase their knowledge and broaden their horizon: *I always find something like that quite cool, because I always ask people how it is at their homes when I have the time because you never stop learning and I always find new cultural aspects, religious aspects, ethnic aspects, and things to get to know.* (P1, L. 32) *It is, yes, interesting to get to know a new culture. This represents an increase in knowledge. It encourages you to question your actions and reflect on yourself.* (P2, L. 34).

To overcome the language barrier, participants exploited diverse opportunities and secured a trustful relationship with their patients. The team evaluated whether someone spoke the respective language to take responsibility for caregiving. Additionally, relatives were used as interpreters. A list of standard questions and a small dictionary from the ward were used. Since employees can hardly cover the entire repertoire of languages, support by interpreting services could be received by nurses, *and when it comes to relevant, even more relevant things, such as psychological problems or explanations, there is a video interpreting service in the house, which you can request. We use that very often*. (P2, L. 26).

Language assistance can be provided through various media and applications. Cell phones enable them to translate sentences quickly and easily*. Everyone has a cell phone, everyone can type in ‘how are you or do you want something to drink or eat’ [long breath], and the device translates it automatically. Reading is something that most people can do, but it also has a voice function. This was a simple strategy.* (P7, L. 44).

Some communication may not be restricted to the linguistic level. Facial expressions and gestures were interpreted in addition to vital signs to obtain indications of the patient’s condition. *Even if language is a barrier, there are still gestures and facial expressions that do not always present themselves as barriers.* (P3, L. 30).

##### Possibilities of the organizations

Healthcare institutions need to coin the term cultural safety and create an overarching awareness of it. Employers should exemplify it and translate it to the mission statement: *Yes, we have a prayer room anyway and that is not the end of culture, and a hospital must stand up for it and that is not yet the case. I know the mission statements of the two hospitals, and there it says, for example, I do not know whether sexual orientation is specifically mentioned. These topics were completely indifferent.* (P1, L. 52).

The nurses suggested holding language courses for employees, building prayer rooms, expanding chaplaincy, and organising cultural festivals. There was a need for easier access to information and introducing a qualified care expert as a contact person, *which shows that they welcome information material in different languages, for example, so that the patient then clearly notices: Oops, they are prepared for me and my culture*. (P9, L. 36).

Education and training are frequently mentioned as a means of improving cultural safety in hospitals. Education on cultural issues was offered routinely, but cultural safety was not covered mostly by the curricula, *which was also part of it for me. With further training and (aah) further training, especially through the introduction of nursing experts, which for me can play a significant role in improving such topics and can play a key role in taking up such topics and bringing them in accordingly to train the team to implement and connect these conceptualisations, disseminate and verbalise, and complete and communicate.* (P3, L. 40).

A multicultural care team has great benefits for patients and the entire health care sector; *therefore, it is enormously useful because I think yes, as culturally diverse as we are in the care team, as diverse are the patients who come.* (P9, L. 22).

Teams with a high degree of diversity had diverse resources ranging from language skills to religious understanding: *We are a multicultural team and I think that can be transferred to the Austrian care landscape accordingly. This means that we have so many resources and so many human, cultural, and religious resources that we can use to provide adequate, humane, goal-oriented, and professional care (aah).* (P3, L. 34).

A diverse nursing team had easier access to the patient, but also the general openness towards foreign cultures increased; *we have a lot of foreign staff who have been here for a long time, for example, from Slovenia, Slovakia, and Croatia, so they are all mixed up, which is why we are a bit more open*. (P6, L. 40).

A team of culturally diverse employees could be better prepared to grasp the importance of cultural safety, since they might have been in unpleasant situations themselves and could share these experiences with their colleagues, *which enriches the whole [team]. Me. That is great. I feel so comfortable.* (P9, L. 22).

##### Proactive involvement of family members

Family members and relatives were enriched in developing a caring relationship. Strong family cohesions were created during visits. *Yes, what is often the case with us is that you can see how the family sticks together; for example, there are always visitors, especially in the palliative setting, how much they care for their relatives. That is a big cohesion, so that is with us Austrians, if I say it now so casually, not so big, as there, the family is written in capital letters. (P6, L. 36).*

The participants should pay attention to integrating and involving families in the care process, *and I have always received positive feedback from these families. So I have noticed that it is less often a single person, but that it was more often a family that was cared for.* (P8, L. 28).

Those patients who had a powerful sense of family could benefit from it as it contributed to recovery: *Well, I do find, [long pause for breath] that one often sees that the visit of the relatives to the patients can already make a difference.* (P7, L. 37).

### Phase 2: nominal group meeting

The most dominant themes of the nominal group interview were ‘communication difficulties,’ ‘lack of knowledge and understanding of how to treat people who do not have sufficient language skills,’ ‘having a different perception of nursing care,’ or ‘expectations towards treatment goals and outcomes,’ which could lead to conflicts.

**Fig. 1:**
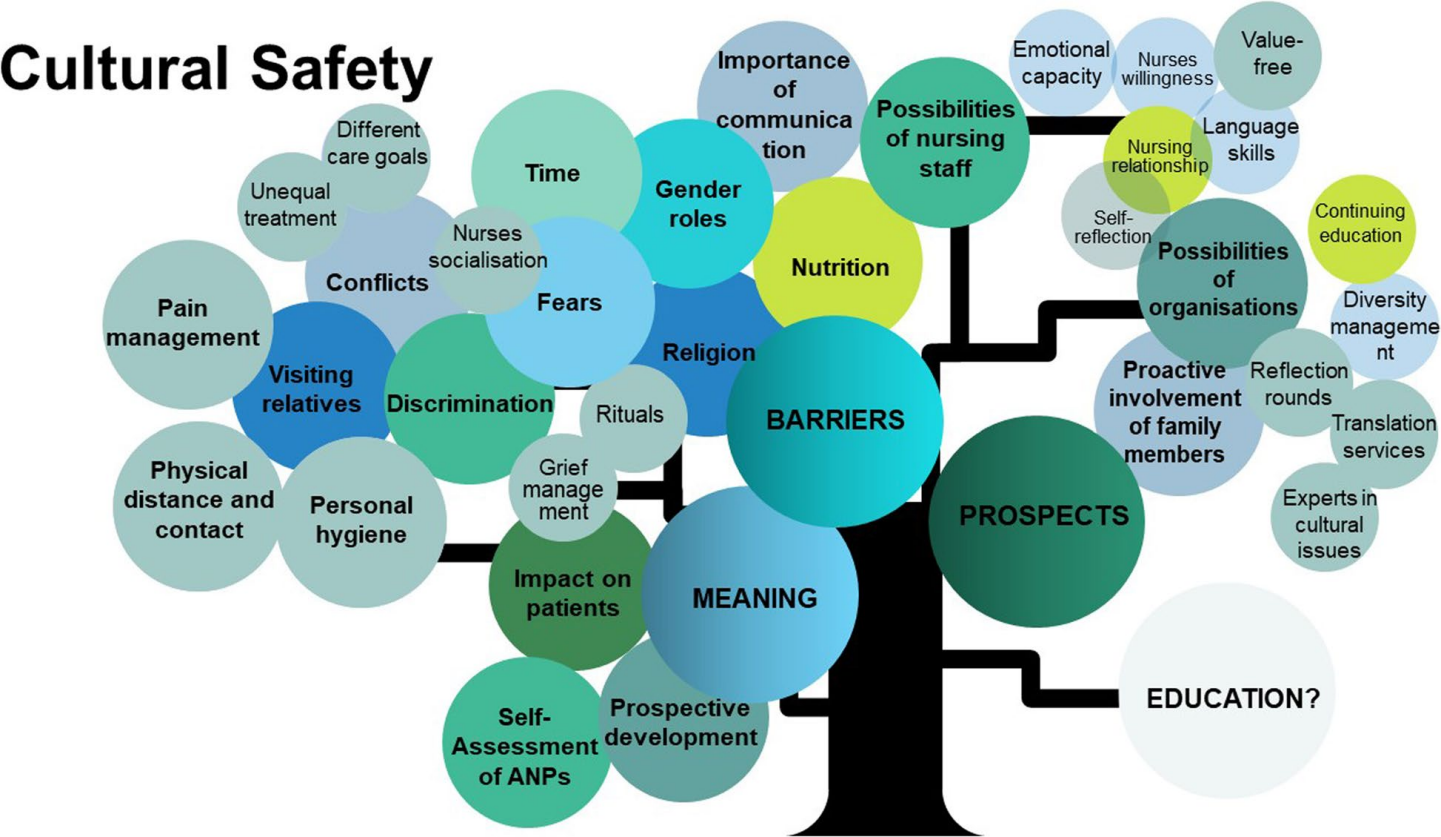
Cultural Safety: meaning, barriers, prospects

After analysing and combining the results of Phases 1 and 2, three themes were developed: ‘physical distance and contact’, ‘pain management (and perception)’, and ‘personal hygiene requirements’. Five subthemes of ‘the socialisation of nurses,’ ‘different care goals,’ and ‘allocations of unequal treatment based on culture,’ were added to the theme ‘conflicts. A lack of knowledge regarding religious rituals and grief management was added to the theme of ‘religion.’ All of these additions constituted the category of barriers to cultural safety. In terms of prospects of cultural safety, the value-free approach was added as a subtheme to the theme ‘possibly of nursing staff’. The possibilities of the organisation were expanded with three subthemes: ‘translation services,’ reflection rounds,’ and ‘introducing experts in cultural safety to the wards.’ The findings are presented as a tree diagram, combining the findings from phases 1 and 2 (Fig. 1).

**Fig. 2:**
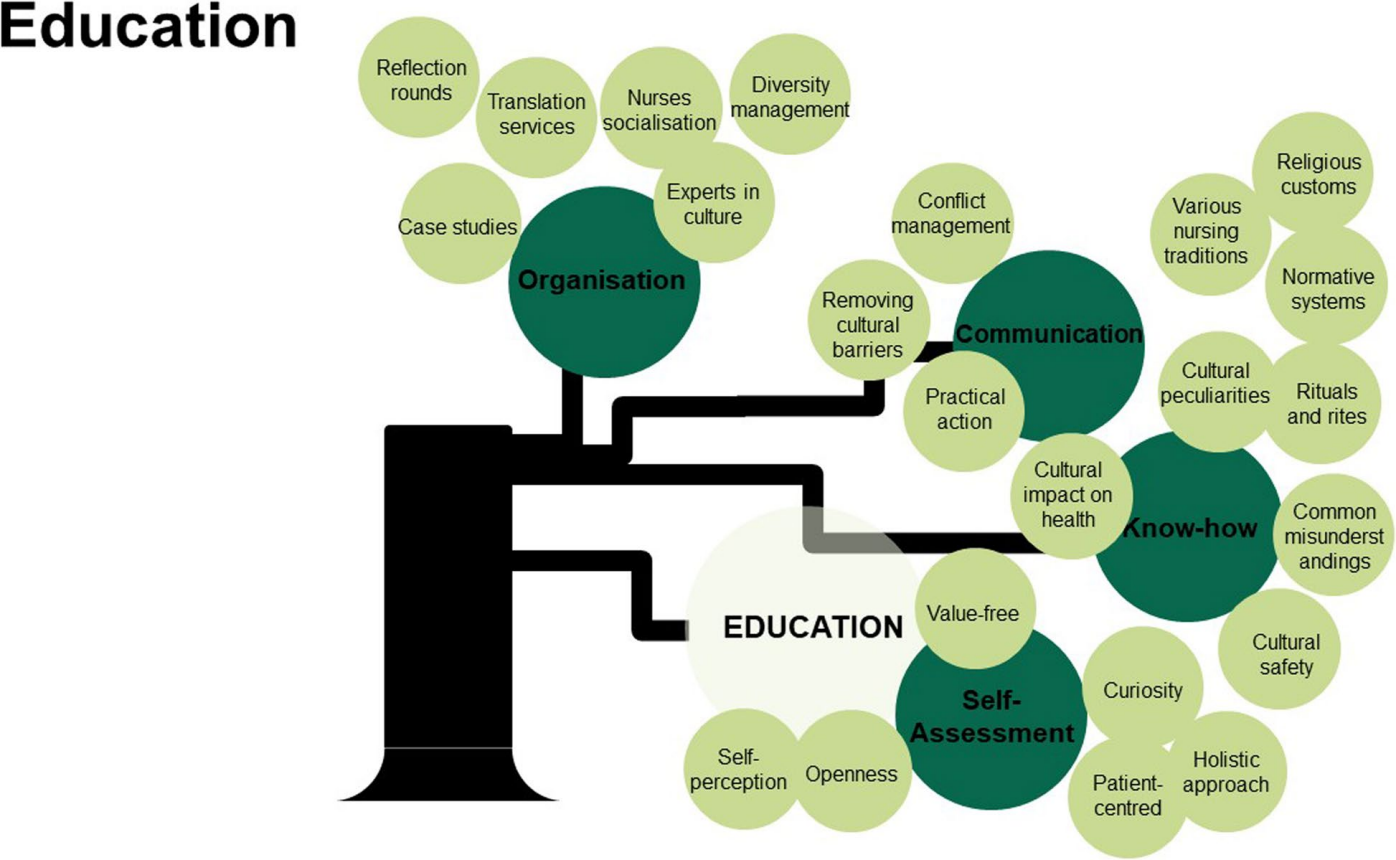
Requirements and expectations toward nursing curriculum and continuing education

All participants in the nominal group pointed out the need for information, knowledge, and continuing education. It became evident that the APNs expected the nursing curriculum and continuous education to increase their knowledge about cultural differences and religious traditions and provide practical communication strategies to overcome cultural barriers. Challenging nurses’ cultural biases and adapted behaviours via self-reflection were suggested less frequently. Organisations are expected to acknowledge the need for cultural safety through diversity management, reflection rounds, and evaluations. Participants’ requirements and expectations towards education are shown in Fig. 2.

## Discussion

In 2016, Bozorgzad et al. wrote: “Healing occurs in a safe milieu, and patients feel safe when service providers view them as whole persons, recognizing the multiple underlying factors that cause illness. Cultural safety can lead to service delivery in this way, but most nurses have no clear understanding of this concept.” [37]. The findings of this study confirm that the APNs do not have a clear understanding of the phenomenon of cultural safety. The admitted APNs were equally ambivalent and lacked knowledge of established concepts, such as transcultural nursing, equity, cultural awareness, cultural safety, or respective competencies. They expected the nursing curriculum and continuous education to increase their cultural competencies, but they did not understand that the concept of cultural safety goes far beyond the acquisition of information, such as religious or dietary needs. Some of the APNs eagerly proposed their definitions of cultural safety, arguing that cultural safety acknowledges the importance of patient-family centred approaches and may help avoid stereotyping patients and their families based on their language, culture, religion, or ethnicity. Similarly, the international literature suggests that approaching the patient, emphasizing on the humanistic aspects of care in terms of hope and life, reducing the feeling of loneliness during hospitalization, paying attention to patients’ needs, sympathizing, and respecting them, and taking their concerns seriously can promote the feeling of safety among hospitalized patients [38].

Heeding cultural safety concerns healthcare staff as much as it concerns patients. Patient safety programs often underestimate the relationship between culture, language, and safety and quality of care [39]. This calls for organizational change that ensures appropriate communication with patients and their families, and supports policies that put in place improvement strategies [40–42], such as reflection rounds or cultural safety briefings at their workplaces [43–45]. Ideally, the education and deployment of nursing experts with a focus on cultural issues would be helpful [46]. Discrimination and even indifference towards patients who do not speak the language may have damaging consequences for patients, affecting their emotional, spiritual and physical wellbeing [47]. Our study findings indicate that patients who were unable to communicate due to poor language skills were systematically left without care. This is a clear example of a lack of access to healthcare and a lack of health equity in healthcare facilities. Taking care of people who do not understand the language or who are poorly educated takes more time and can be emotionally draining. Compensation and support should be offered to nurses taking over a mediating role. Culturally diverse nurses are considered assets in terms of language skills and cultural competencies when cultural clashes emerge. However, in our study, culturally diverse APNs admitted their familiarity with prejudices, discrimination, and racism, which was presented as a taboo topic. Whereas, patient safety culture and the second victim phenomenon has been described as causing distress in nurses [48], this study emphasises the first victim experience in culturally diverse nurses.

Power represents an essential distinction between cultural competence and cultural safety [15, 16, 37]. Healthcare providers should reflect on interpersonal power differences and acknowledge the power differences between nurses and patients. The topic of power was not discussed in our explorative study, which could be because of the traditional development of nursing in German-speaking countries. Nursing as an independent and academic discipline has developed very slowly in Austria, Germany, and Switzerland, compared to the international context. In Austria, basic training in nursing was shifted to the tertiary level with a change in the law in 2016 [49], university and academic training in nursing is still a much-discussed topic. The service to the patient according to the principle of Christian charity is in the foreground, strictly maintaining the attitude that the doctors order the interventions and the nurses perform them. In many cases, there is still a lack of awareness of one’s own professional role and the power it entails in the relationship between patient and nurse.

The issue of visiting relatives divided the APNs. Large families were perceived as a source of support and taking on caring responsibilities, but others saw it as a hindrance due to noise and disturbance. Despite ongoing criticism of the exhaustion of family members as interpreters [50], relatives were perceived as helpful in overcoming communication barriers. Supporting relational, culturally safe care focuses on building the conversation, engaging the family, closing the conversation gap and improving health literacy to reduce power imbalances [15]. Some nurses found it personally and professionally fulfilling to be proactive in informing, involving and communicating with family members to better understand the patient’s needs.

Nurses need to evaluate and question their cultural backgrounds and adapt their behaviours rather than pushing for the mere acquisition of competencies in different cultures and religions [16]. APNs admitted that they do not provide culturally safe care, but unanimously asserted that they strive to provide such care. They cited several characteristics of safe patient care in terms of removing language barriers and overcoming misunderstandings as the first step in building a professional nursing relationship. It became clear that reflecting on one’s own values, attitudes, assumptions, prejudices and stereotypes is essential for culturally safe care. Nurses mentioned that self-reflection must be an ongoing process to ensure culturally safe care on a continuous basis. Our study described many components that can contribute to improving cultural safety. Previously introduced concepts such as transcultural care, cultural sensitivity, cultural awareness and competencies were interpreted as prerequisites for cultural safety. Other important aspects were professional flexibility, openness and tolerance of nurses in caring for culturally diverse patients and their families. In addition, APNs argued that an atmosphere free of intimidation and fear promotes overall health [38, 48]. Accordingly, the concept of cultural safety offers the possibility of ensuring better access to healthcare for culturally diverse patients and their families in pluralistic societies [51].

### Strengths and limitations to the study

This is the first study on cultural safety in a European context, which underlines the novelty of the research topic. The simultaneous use of interviews and nominal groups for data collection from APNs working in health facilities in three countries helped to improve the variation and depth of data collection and certainly represents our research strength. In terms of informativeness, this qualitative study benefited from it through the use of the nominal group meeting in Phase 2 to validate the findings of Phase 1 and contribute to the development of new knowledge.

Online interviews cannot replace face-to-face interactions, but they offer a viable alternative [52]. A comparison of online interviews and face-to-face interviews shows that participants are more open and expressive online, while maintaining a relationship requires more effort [53].

## Conclusions

Communication difficulties, insufficient language skills, different perceptions and expectations of care and treatment outcomes were mentioned as important aspects of cultural safety in German-speaking health facilities. There is a need for self-reflection sessions and translation services for nurses responsible for the care of patients with diverse cultural backgrounds in order to improve equality and the feeling of safety during the hospital stay. In addition, the presence of prejudice, discrimination and even racism in the healthcare culture should be assessed and remedial strategies developed and implemented using diversity management and leadership. Education and training are needed to bridge the gap between the perceived educational needs of nurses and the requirements of safe care, and to address challenging personal behaviours in the multicultural healthcare environment. Future studies should examine the impact of cultural safety improvement programmes on patient care outcomes.

## Data Availability

The anonymous data and the analysis process used in this study are available from the corresponding author upon reasonable request.

## References

[1] UN Office of the High Commissioner for Human Rights (2008). Fact Sheet No. 31, The Right to Health.

[2] International Council of Nurses (2021). The ICN Code of Ethics for Nurses.

[3] Papps E, Ramsden I (1996). Cultural Safety in Nursing: the New Zealand Experience. Int J Qual Health Care.

[4] Sy A (2013). Who Defines Culturally Acceptable Health Access? Universal rights, healthcare politics and the problems of two Mbya-Guarani communities in the Misiones Province, Argentina. Health Cult Soc.

[5] National Committee for Quality Assurance (2016). A Practical Guide to Implementing the National CLAS Standards: For Racial, Ethnic and Linguistic Minorities, People with Disabilities and Sexual and Gender Minorities.

[6] European Council (2018). A New European Agenda for Culture.

[7] Power T, Geia L, Adams K, Drummond A, Saunders V, Stuart L, Deravin L, Tuala M, Roe Y, Sherwood J, Rowe Minniss F, West R (2021). Beyond 2020: addressing racism through transformative indigenous health and cultural safety education. J Clin Nurs.

[8] Perry A, Woodland L, Brunero S (2015). eSimulation: a novel approach to enhancing cultural competence within a health care organisation. Nurse Educ Pract.

[9] Richardson S, Williams T (2007). Why is cultural safety essential in health care?. Med Law.

[10] Bundesrepublik für Migration, Flüchtlinge und Integration (2009). Memorandum für eine kultursensible Altenhilfe Ein Beitrag zur Interkulturellen Öffnung am Beispiel der Altenpflege.

[11] World Health Organization (2019). Declaration of Astana: Global Conference on Primary Health Care.

[12] Helman, C.G. Culture, Health and Illness. 4th Ed. London: Hodder Arnold. 2000.

[13] Paal, P. Culturally sensitive palliative care research: what should we do with ‘those people’, or what should we do with ourselves? In: K. Kuehlmeyer, C. Klingler & R. Huxtable (eds.) Ethical, Legal and Social Aspects of Healthcare for Migrants: Perspectives from the UK and Germany. London&New York: Routledge. 2018. p. 162–75.

[14] Cubillos, F.A. Pflege in einer internationalen und transkulturellen Gesellschaft. In: A. Merx (ed.) Charta der Vielfalt: WELTOFFEN = ZUKUNFTSFÄHIG?! Diversity Management & Internationalität (pp. 62–64). Retrieved May 2, 2021, from: https://www.charta-der-vielfalt.de/fileadmin/user_upload/Studien_Publikationen_Charta/Weltoffen_Zukunftsf%C3%A4hig_CdV-Dossier.pdf

[15] Curtis E, Jones R, Tipene-Leach D, Walker C, Loring B, Paine SJ, Reid P (2019). Why cultural safety rather than cultural competency is required to achieve health equity: a literature review and recommended definition. Int J Equity Health.

[16] Wilson C, Crawford K, Adams K (2022). Translation to practice of cultural safety education in nursing and midwifery: A realist review. Nurse Educ Today.

[17] Dicuccio MH (2015). The Relationship Between Patient Safety Culture and Patient Outcomes: A Systematic Review. J Patient Saf.

[18] Pumar-Méndez MJ, Attree M, Wakefield A (2014). Methodological aspects in the assessment of safety culture in the hospital setting: A review of the literature. Nurse Educ Today.

[19] International Council of Nurses (2008). The scope of practice, standards and competencies of the advanced practice nurse. ICN Regulatory Series.

[20] International Council of Nurses. Guidelines on advanced practice nursing 2020. International Council of Nurses (ICN). 2020. Retrieved February 10, 2022, from: https://www.icn.ch/system/files/documents/2020-04/ICN_APN%20Report_EN_WEB.pdf

[21] Hamric AB, Spross JA, Hanson CM (2008). Advanced Practice Nursing: An Integrative Approach.

[22] Glarcher M, Lex KM (2020). Advanced Nursing Practice in Austria under consideration of outcome measurement. Zeitschrift für Evidenz, Fortbildung und Qualität im Gesundheitswesen (ZEFQ).

[23] Spichiger E, Zumstein-Shaha M, Schubert M, Herrmann L (2018). Gezielte Entwicklung von Advanced Practice Nurse-Rollen für spezifische Patient(inn)engruppen in einem Schweizer Universitätsspital. Pflege.

[24] Bachner F, Bobek J, Habimana K, Ladurner J, Lepuschütz L, Ostermann H, Rainer L, Schmidt AE, Zuba M, Quentin W, Winkelmann J (2018). Austria: Health system review. Health Syst Trans.

[25] Weiss, S., Ditto, M., Füszl, S., Lanske, P., Lust, A., Oberleitner-Tschan, C., Wenda, S. Healthcare professions in Austria. Federal Ministry of Health and Women’s Affairs, Division II, 2017. Retrieved May 23, 2022, form: https://broschuerenservice.sozialministerium.at/Home/Download?publicationId=600

[26] Schäfer, D. Die Zukunft der „Advanced Practice Nurse“ in Österreich. Pflege Professionell, 2018. 25.06.2018, Retrieved May 23, 2022, from: https://pflege-professionell.at/die-zukunft-der-advanced-practice-nurse-in-oesterreich

[27] DeutscherBerufsverbandfürPflegeberufe - DBfKBundesverbande.V. (2019). Advanced Practice Nursing Pflegerische Expertise für eine leistungsfähige Gesundheitsversorgung.

[28] Lamnek S, Krell, C. Qualitative Sozialforschung: Mit Online-Materialien. 6th Ed. Psychologie Verlagsunion. Weinheim: Peltz; 2016.

[29] Flick U, Kardoff E, Steinke I. Qualitative Forschung. Hamburg: Rowohlt Taschenbuchverlag; 2019.

[30] Malterud K, Siersma VD, Guassora AD (2016). Sample Size in Qualitative Interview Studies: Guided by Information Power. Qual Health Res.

[31] International Council of Nurses (2009). Definition and Characteristics of the Role.

[32] Ammouri A, Tailakh A, Muliira J, Geethakrishnan R, Al Kindi S (2015). Patient safety culture among nurses. Int Nurs Rev.

[33] Kuckartz, U. Qualitative Inhaltsanalyse. Methoden, Praxis, Computerunterstützung. Weinheim und Basel: Beltz Juventa; 2016.

[34] Clarke V, Braun V (2017). Thematic analysis. J Posit Psychol.

[35] Vaismoradi M, Jones J, Turunen H, Snelgrove S (2016). Theme development in qualitative content analysis and thematic analysis. J Nurs Educ Pract.

[36] Mcmillan S, Kelly F, Sav A, Kendall E, King MA, Whitty JA, Wheeler AJ (2014). Using the nominal group technique: How to analyse across multiple groups. Health Serv Outcomes Res Methodol.

[37] Bozorgzad P, Negarandeh R, Raiesifar A, Poortaghi S (2016). Cultural Safety: An Evolutionary Concept Analysis. Holist Nurs Pract.

[38] Vaismoradi M, Salsali M, Turunen H, Bondas T (2011). Patients’ understandings and feelings of safety during hospitalization in Iran: A qualitative study. Nurs Health Sci.

[39] Johnstone M-J, Kanitsaki O (2006). Culture, language, and patient safety: making the link. Int J Qual Health Care.

[40] Hudelson P, Vilpert S (2009). Overcoming language barriers with foreign-language speaking patients: a survey to investigate intra-hospital variation in attitudes and practices. BMC Health Serv Res.

[41] Verbakel NJ, Langelaan M, Verheij TJM, Wagner C, Zwart DL (2016). Improving Patient Safety Culture in Primary Care: A Systematic Review. J Patient Saf.

[42] Gurková E, Zeleníková R, Friganovic A, Uchmanowicz I, Jarošová D, Papastavrou E, Žiaková K (2020). Hospital safety climate from nurses’ perspective in four European countries. Int Nurs Rev.

[43] Flanagan E, Chadwick R, Goodrich J, Ford C, Wickens R (2020). Reflection for all healthcare staff: A national evaluation of Schwartz Rounds. J Interprof Care.

[44] Ryan S, Ward M, Vaughan D, Murray B, Zena M, O’Connor T, Nugent L, Patton D (2018). Do safety briefings improve patient safety in the acute hospital setting? A systematic review. J Adv Nurs.

[45] Weaver SJ, Lubomksi LH, Wilson RF, Pfoh ER, Martinez KA, Dy SM (2013). Promoting a Culture of Safety as a Patient Safety Strategy A Systematic Review. Ann Intern Med.

[46] Salmela S, Koskinen C, Eriksson K (2017). Nurse leaders as managers of ethically sustainable caring cultures. J Adv Nurs.

[47] Schrank B, Rumpold T, Amering M, Masel EK, Watzke H, Schur S (2017). Pushing boundaries — culture-sensitive care in oncology and palliative care: a qualitative study. Psychooncology.

[48] Quillivan RR, Burlison JD, Browne EK, Scott SD, Hoffman JM (2016). Patient Safety Culture and the Second Victim Phenomenon: Connecting Culture to Staff Distress in Nurses. Jt Comm J Qual Patient Saf.

[49] Schaeffer, D. Nursing science and research: quo vadis? In E. Seidl & I. Walter (Eds.), Current nursing research. Studies-Comments-Reports. Pflegewissenschaft heute, 7. Wien, München, Bern: Maudrich. 2002. p. 129–50.

[50] Barclay JS, Blackhall LJ, Tulsky JA (2007). Communication Strategies and Cultural Issues in the Delivery of Bad News. J Palliat Med.

[51] Latif AS (2020). The Importance of Understanding Social and Cultural Norms in Delivering Quality Health Care—A Personal Experience Commentary. Trop Med Infect Dis.

[52] LaIacono V, Symonds P, Brown DHK (2016). Skype as a Tool for Qualitative Research Interviews. Sociol Res Online.

[53] Gray LM, Wong-Wylie G, Rempel GR, Cook K (2020). Expanding Qualitative Research Interviewing Strategies: Zoom Video Communications. Qual Rep.

